# Development of rhabdomyolysis in a child after norovirus gastroenteritis

**DOI:** 10.1186/s12887-016-0720-9

**Published:** 2016-11-04

**Authors:** Tomohiro Nishio, Ryoko Yonetani, Eisuke Ito, Makiko Yoneta, Yoshihiro Maruo, Tokiko Yoshida, Tohru Sugimoto

**Affiliations:** 1 Department of Pediatrics, Saiseikai Shiga Hospital, Social Welfare Organization, Saiseikai Imperial Gift Foundation Inc, Ritto, Shiga Japan; 2 Shiga Prefectural Institute of Public Health, Otsu, Shiga Japan; 3 Department of Pediatrics, Shiga University of Medical Science, Otsu, Shiga Japan; 4 Shiga Saiseikai Nursing School, Social Welfare Organization, Saiseikai Imperial Gift, Foundation Inc, Ritto, Shiga 520-3046 Japan

**Keywords:** Gastroenteritis, Norovirus, Epidemiology, Rhabdomyolysis, Dysstasia

## Abstract

**Background:**

In children, the most significant cause of rhabdomyolysis or muscle breakdown is viral infection. However, there are no reports that norovirus, a gastroenteric virus that commonly infects children, specifically causes rhabdomyolysis. Here, we report the first pediatric case of norovirus-associated rhabdomyolysis.

**Case presentation:**

The patient, a 2-year-old boy with fever, diarrhea, and vomiting, was referred to our hospital with dysstasia and transaminitis. He was diagnosed with rhabdomyolysis. Additionally, norovirus genogroup GII was detected from stool samples by real-time quantitative reverse transcription Polymerase Chain Reaction, and thereafter, the norovirus GII.4 variant was identified.

**Conclusion:**

However, the association between rhabdomyolysis and the isolated norovirus variant was not clarified. After treatment the patient recovered without renal failure or disseminated intravascular coagulation. Rhabdomyolysis is a disease for which there is a need for early detection and treatment. If abnormal posture or muscle weakness is observed during the course of gastroenteritis, blood and urinary tests should be performed to rule out rhabdomyolysis.

## Background

Due to decreased incidence of rotavirus as the result of the rotavirus vaccine, norovirus has become the leading cause of acute gastroenteritis in children less than 2 years old in Japan as well as in the USA [1, 2]. Rhabdomyolysis, muscle breakdown, in children is caused by acute viral infections such as influenza A/B, coxsackievirus, and Epstein-Barr virus [3]. In up to 40 % of children with rhabdomyolysis, renal failure is reported to develop. Delay in the detection of rhabdomyolysis may increase the risk of development of acute renal failure [4]. Therefore, early diagnosis and initiation of appropriate treatments for rhabdomyolysis is crucial. However, infants and young children are unable to clearly complain of these symptoms and thus require close examination. Here we report the first case describing rhabdomyolysis in a norovirus-infected child.

## Case presentation

**Fig. 1:**
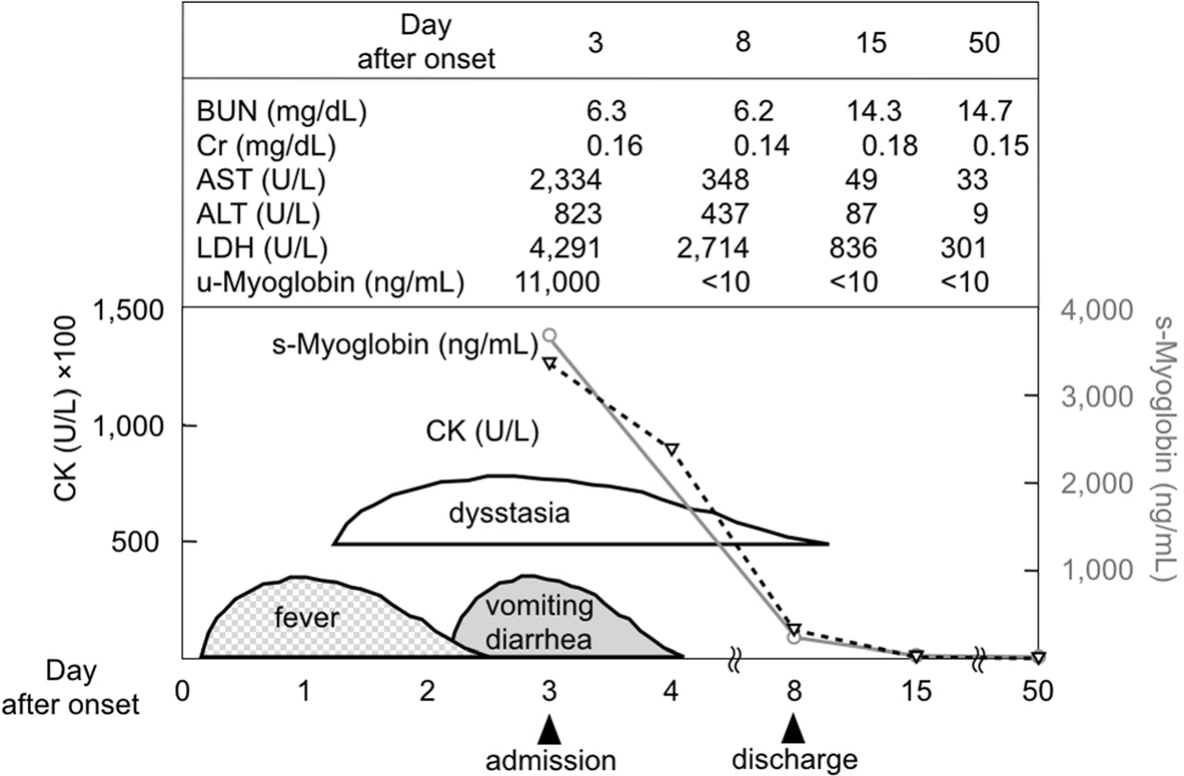
Representative clinical manifestation and laboratory data

The patient, an ethnically Japanese 2-year-old boy, was referred to our hospital with dysstasia and transaminitis. In Fig. 1, representative clinical manifestation and laboratory data are shown. The boy was well since birth until the onset of illness. On the first day, he developed a fever and was treated with acetaminophen suppositories. On the second day after onset of illness, his family took him to a nearby clinic because of the persistent fever, along with diarrhea and vomiting. Additionally, the child was unable to stand. A rapid diagnostic test for influenza A and B viral antigens yielded negative results. Although the fever subsided on the third day, the child was still unable to stand and was taken to the clinic again (Fig. 1). Physical examination showed muscle weakness in the lower extremities. Blood examination revealed high levels of transaminases, indicative of rhabdomyolysis. Thus, the child was admitted to our hospital.

On admission (11 January 2012), there was no fever, and the child was awake and alert. Vital signs were normal, except for slight tachycardia. Examination of the chest and abdomen revealed no abnormalities. There was no swelling, warmth, or tenderness of the joints or muscles of the extremities. Muscle weakness and blunted deep tendon reflexes were noted in the lower limbs. The child had been able to walk independently prior to the onset of this illness; however, he was now unable to stand up or maintain a stable sitting position (Fig. 1).

Normal white blood cell (WBC) and slight increase of C-reactive protein were observed. Serum electrolyte levels were normal. Blood urea nitrogen (BUN) and serum creatinine (Cr) were within normal limits. Normal serum levels of bilirubin, cholinesterase, and ammonia were observed, as well as normal blood levels of glucose lactate and acidosis. The child also presented a normal coagulation profile. Serum levels of aspartate aminotransferase (AST) (2, 334 U/L), alanine aminotransferase (ALT) (823 U/L), lactate dehydrogenase (LDH) (4, 291 U/L) and creatine kinase (CK) (100, 637 U/L) were elevated (Fig. 1). The levels of the three main isoforms of serum CK (MM, MB, and BB) were 100, 0, and 0 %, respectively, indicating a muscular rather than cardiac origin of rhabdomyolysis. High serum and urinary levels of myoglobin (3, 680 ng/mL and 11, 000 ng/mL, respectively) were also observed (Fig. 1). Qualitative urinalysis yielded a positive occult blood test; however, the urinary sediment revealed no red blood cells.

To understand the viral cause of the presented patient’s symptoms, we systematically tested for a battery of viruses. From a stool sample taken upon admission, the norovirus genogroup was determined by real-time quantitative reverse transcription (RT)-PCR [5]. For the detection of norovirus GI, primers COG1F and COG1R and probes RING1-TP(a) and RING1-TP(b) were used. For the detection of norovirus GII, primers COG2F/ALPF and COG2R, and probe RING2AL-TP were used [5]. Norovirus GII was moderately detected (3.7× 10^7^ copies/g feces); however, norovirus GI was not. To analyze the genotype of the detected norovirus GII strain, the partial capsid gene products were amplified by PCR using the primer sets COG2F/G2-SKR (387 bp) [5] (http://www.mhlw.go.jp/topics/syokuchu/kanren/kanshi/031105-1a.pdf).

The genotype of the norovirus was characterized by direct nucleotide sequencing of the partial capsid gene and was then identified with norovirus GII.4 Den_Haag_2006b variant according to the Norovirus Genotyping Tool Version 1.0 (http://www.rivm.nl/mpf/norovirus/typingtool).

A pharynx swab taken upon admission was examined for enteroviral subgroups (coxsackieviruses, echoviruses, and enteroviruses) by RT-PCR; however, none was detected. A stool sample taken on the fifth day after illness onset, examined by the latex agglutination test, was negative for both rotavirus and adenovirus infections. Additionally, results of serum tests for HBs antigen and HCV antibody were negative. Serum test results for IgM antibodies against Epstein-Barr virus, herpes simplex virus, and cytomegalovirus were negative. Soon after admission, we attempted to isolate pathogenic bacteria from a stool sample by conventional methods; however, none was found. From these bacterial and viral laboratory results, the most plausible causative pathogen for rhabdomyolysis was norovirus.

Treatment was initiated with intravenous administration of bicarbonate Ringer’s solution and oral administration of the diuretic acetazolamide. On the sixth day after onset of illness, we found that the therapies could be discontinued without the development of renal failure or disseminated intravascular coagulation. On the seventh day, the deep tendon reflexes became normal, and the child moved his legs often in the supine position. On the eighth day, serum and urinary myoglobin levels had improved to 238 ng/mL and <10 ng/mL, respectively. The patient was able to maintain a stable sitting position and was discharged from the hospital (Fig. 1).

## Discussion

Norovirus infection is known to be associated with various complications, including rash, convulsive attacks, encephalopathy, ileus, invagination, hemophagocytic syndrome, and metabolic acidosis. However, the association with norovirus infection and rhabdomyolysis has never been reported. Mannix et al. investigated 191 children with rhabdomyolysis and reported that the most frequent cause was viral myositis (38.2 %). Other causes included trauma (25.7 %), connective tissue diseases (5.2 %), drug overdose (4.2 %), exercise (4.2 %), and metabolic disorders (3.7 %) [6]. Rhabdomyolysis has been reported to develop following various viral infections [3]. In our present case, influenza virus, enteroviral subgroups, rotavirus, adenovirus, hepatitis B virus, hepatitis C virus, Epstein-Barr virus, herpes simplex virus, and cytomegalovirus were ruled out by our laboratory data. A search of the literature on rhabdomyolysis associated with viral gastroenteritis revealed only one report of rhabdomyolysis associated with rotavirus infection [7]. This report stated that the patient developed encephalopathy, rhabdomyolysis, hypotensive shock and severe electrolyte abnormalities [7].

In children with organic and fatty acid metabolic disorders, rhabdomyolysis can be triggered by infection, stress, and starvation [8]. However, the patient’s levels of blood carnitine and urinary organic acids on admission were normal (data not shown); therefore, organic and fatty acid metabolic disorders (but not all metabolic diseases) can be excluded. Except for the period of hospital admission at age 2, the patient has been well from birth until his present age of 6 years old. The vital signs at the first visit to our hospital were stable, making it unlikely that the patient had rhabdomyolysis, which is associated with inborn errors of metabolism and mitochondrial disease. There was no history of trauma, severe exercise or heat stress. The laboratory test results upon admission showed no electrolyte abnormalities or severe inflammatory reactions. There was no evidence of alcohol consumption or drug overdose. These observations strongly suggested that the rhabdomyolysis was caused by viral myositis.

From 2009 to 2013, the incidence of viral acute gastroenteritis among 2,381 Japanese pediatric patients was examined by Thongprachum et al. [9]. Norovirus was predominantly detected (39.3 %), whereas the prevalence of rotavirus, human parechovirus, enterovirus, and adenovirus was 20.1, 6.6, 6.1, and 5.6 %, respectively [9]. The molecular epidemiology of norovirus genotypes in this population were also analyzed by Thongprachum et al. [10]. Noroviruses are highly diverse and can be divided into five genogroups (GI, II, III, IV and V) [9]. Norovirus GII, which is the most common genogroup of acute gastroenteritis, was detected in our patient’s fecal specimen. When this patient was admitted (January 2012), the norovirus GII.4 genotype Den_Haag_2006b variant (43.2 %) was the most common genotype, and then the norovirus GII.4 genotype New_Orleans_2009 variant (17.8 %) co-circulated until March 2012 based on work by Thongprachum et al. [10]. Subsequently, they were displaced by the norovirus GII.4 genotype Sydney_2012 variant [10]. We isolated the norovirus genotype GII.4 Den_Haag_2006b variant from our patient upon his admission, which corroborates this epidemiological report [10].

## Conclusion

This is the first plausible pediatric patient with norovirus and rhabdomyolysis development. The association between rhabdomyolysis, the norovirus GII.4 genotype Den_Haag_2006b variant, and the antigenicity of this GII.4 variant against muscle cells (and thus being responsible for rhabdomyolysis) remains obscure and is worthy of investigation.

## Abbreviations

ALT: Alanine aminotransferase
AST: Aspartate aminotransferase
BUN: Blood urea nitrogen
CK: Creatine kinase
Cr: Creatinine
LDH: Lactate dehydrogenase
PCR: Polymerase chain reaction
RT: Reverse transcription
WBC: White blood cell
